# Molecular pathology of primary aldosteronism and hypercortisolism: Impact on adrenal surgery

**DOI:** 10.1016/j.isci.2026.114910

**Published:** 2026-02-05

**Authors:** Tobias Carling, Constantine A. Stratakis, Fabio R. Faucz, C. Christofer Juhlin

**Affiliations:** 1 Carling Adrenal Center, Tampa, FL, USA; 2 Hospital for Endocrine Surgery Research Center, HCA Florida Healthcare, HCA Healthcare Research Institute, Tampa, FL, USA; 3 ASTREA Health & DIGENIA Human Genetics & Precision Medicine, Athens, Greece; 4 Human Genetics & Precision Medicine, IMBB, FORTH, Heraklion, Greece; 5 Pediatrics, University of Athens Medical School, Athens, Greece; 6 Department of Pathology and Cancer Diagnostics, Karolinska University Hospital, Stockholm, Sweden; 7 Department of Oncology-Pathology, Karolinska Institutet, Stockholm, Sweden

**Keywords:** Health sciences, Medicine, Medical specialty, Surgery

## Abstract

Recent advances in molecular pathology have elucidated the genetic underpinnings of benign aldosterone- and cortisol-producing adrenal lesions, identifying genes encoding mainly potassium and voltage-gated calcium channels in primary aldosteronism (PA), and *PRKACA*, *PRKAR1A*, *ARMC5* in hypercortisolism. These insights are increasingly guiding precise, function-preserving surgery, such as mini back scope adrenalectomy (MBSA) at high-volume centers with reduced morbidity and risk of adrenal insufficiency, even in complex bilateral cases. Postoperative pathological analysis assessing hormone secretion and genetics provides prognostic information and directs further therapy, whether medical or surgical. A modern approach to PA and hypercortisolism, influenced by an enhanced understanding of the molecular pathophysiology, is likely to improve outcomes and expand adrenalectomy usage and indications as the morbidity and mortality associated with these endocrinopathies are increasingly recognized.

## Graphical abstract

**Figure:**
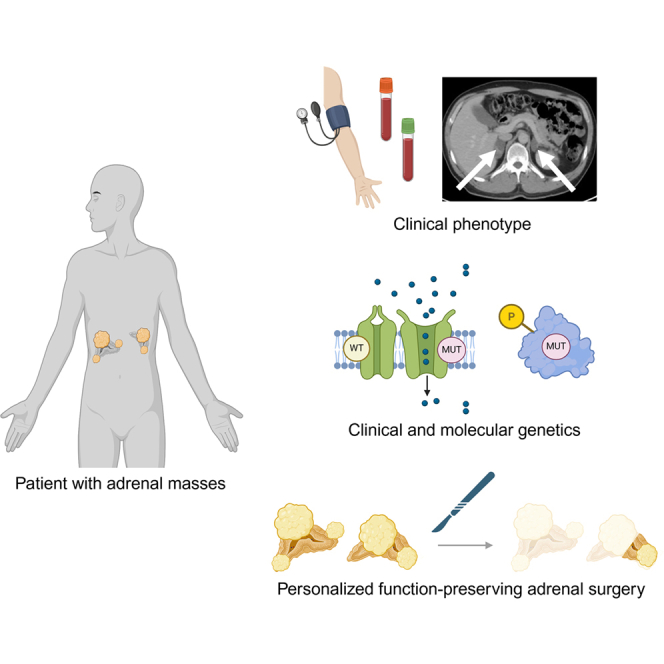

## Introduction

The adrenal glands, situated atop the kidneys, are pivotal endocrine organs regulating a constellation of physiological processes through hormone secretion. The adrenal cortex, its outer domain, produces aldosterone, a mineralocorticoid governing sodium-potassium balance and blood pressure, and cortisol, a glucocorticoid modulating metabolism, immune responses, and stress adaptation.^1^^,^^2^ Dysregulated hypersecretion of these hormones precipitates profound clinical consequences. Primary aldosteronism (PA), the leading cause of secondary hypertension, arises from autonomous aldosterone overproduction, driving resistant hypertension, hypokalemia, and heightened cardiovascular morbidity and mortality (reviewed in Reincke et al., 2021).^1^ Similarly, benign ACTH-independent adrenal hypercortisolism (AHC), a subset of Cushing syndrome stemming from adrenal lesions, manifests as excessive cortisol production, resulting in metabolic derangements, obesity, osteoporosis, cardiovascular complications, and neuropsychiatric disturbances (reviewed in Lacroix et al., 2015).^3^ Surgical removal of the adrenal tumors or hyperplastic nodules of hormone-hypersecreting cells is the most efficacious and only curative treatment, but despite transformative advances in adrenal surgery, propelled by minimally invasive techniques, aldosterone- and cortisol-producing adrenal lesions remain highly underdiagnosed and undertreated.^1^^,^^4^ Estimates suggest that fewer than 1% of PA cases, depending on diagnostic criteria, are identified and thoroughly evaluated,^5^ with even fewer patients offered adrenalectomy, the sole curative option.^6^^,^^7^^,^^8^ A parallel diagnostic and therapeutic gap exists in AHC, particularly in its subclinical form, known as mild autonomous cortisol secretion (MACS).^4^^,^^9^ Barriers to optimal care include low clinical suspicion, labor-intensive and imperfect biochemical diagnostic assays, complex preoperative evaluations, uncertainty in patient selection for surgery, and limited access to high-volume adrenal surgeons.^5^^,^^6^

A detailed exploration of PA and AHC diagnostic and screening strategies lies beyond this review’s scope (reviewed in Adler et al., 2025 for PA; Lacroix et al., 2015 for AHC).^3^^,^^10^ Similarly, pharmacological management is not covered here, but targeted therapies for PA (reviewed in Rossi et al., 2024 and Lechner et al., 2019)^11^^,^^12^ and AHC (reviewed in Fleseriu et al., 2025; Lacroix et al., 2015; and Reincke and Fleseriu, 2023)^2^^,^^3^^,^^13^ play a vital role in specific scenarios: (1) patients unfit for general anesthesia, (2) preoperative management of severe hormone excess, (3) interim treatment for those with morbid obesity awaiting surgery, (4) control of persistent or recurrent disease after adrenalectomy when further surgery is not viable, and (5) care for patients with limited life expectancy. Furthermore, there exists some controversy regarding the extent to which PA and AHC drive morbidity and mortality, and the degree to which adrenalectomy reverses these outcomes (discussed in Chen et al., 2022; Clayton, 2022; Farah et al., 2025; Gkaniatsa et al., 2023; and Prete & Bancos, 2024).^14^^,^^15^^,^^16^^,^^17^^,^^18^ The literature addressing these questions is constrained by insufficient data on the biochemical severity and duration of the disease prior to adrenalectomy, challenges in controlling for common comorbidities such as essential hypertension, obesity, and diabetes, reliance on retrospective study designs, and meta-analyses of primarily observational studies. Nonetheless, accumulating evidence suggests significant improvements in both morbidity and mortality after adrenalectomy, but a detailed exploration of this topic falls outside the focus of this review article.

Recent molecular characterization of aldosterone- and cortisol-producing adrenal lesions has unveiled critical genetic and epigenetic drivers, yet these discoveries have not fully translated into clinical practice. Total unilateral adrenalectomy remains the default surgical approach in many leading institutions, often leading to reluctance in addressing bilateral disease.^6^^,^^19^ This review elucidates the molecular and pathological underpinnings of these adrenal tumor disorders and explores how these insights inform minimally invasive, function-preserving adrenalectomy techniques, enabling the precise resection of hormone-producing lesions while preserving normal hormonal function, thereby mitigating the risk of adrenal insufficiency (hypocortisolism), even in complex bilateral cases.^6^^,^^19^^,^^20^^,^^21^^,^^22^

## Molecular pathology of primary aldosteronism

The most frequent cause of secondary hypertension, PA, is caused by autonomous aldosterone production, from unifocal (single) or multifocal tumors or rarely hyperplasia, which results in hypertension, hypokalemia, and sodium retention. The pathophysiology of PA has been elucidated by molecular genetics, which has clarified the processes underlying both familial and sporadic forms of the disease. Pathologically, PA is caused by various subtypes, including uni- and bilateral aldosterone-producing adenoma (APA and Bi-APA, respectively; size >1 cm), aldosterone-producing nodule (APN; size <1 cm, visible on conventional histological exam using both hematoxylin-eosin (HE) staining, and immunohistochemical (IHC) staining for *CYP11B2*; aldosterone synthase), and aldosterone-producing micronodule (APM; size <1 cm, not visible using HE staining, but seen on IHC staining, previously termed aldosterone-producing cell clusters). Both APNs and APMs may occur as multiple lesions (MAPN/MAPM), and like aldosterone-producing diffuse hyperplasia (APDH), are bilateral.^23^ Each pathological subtype displays distinct molecular and genetic signatures contributing to its development. Multiple aldosterone-producing lesions can coexist, and incidental nonfunctioning adenomas may obscure the aldosterone source.^24^

**Figure 1:**
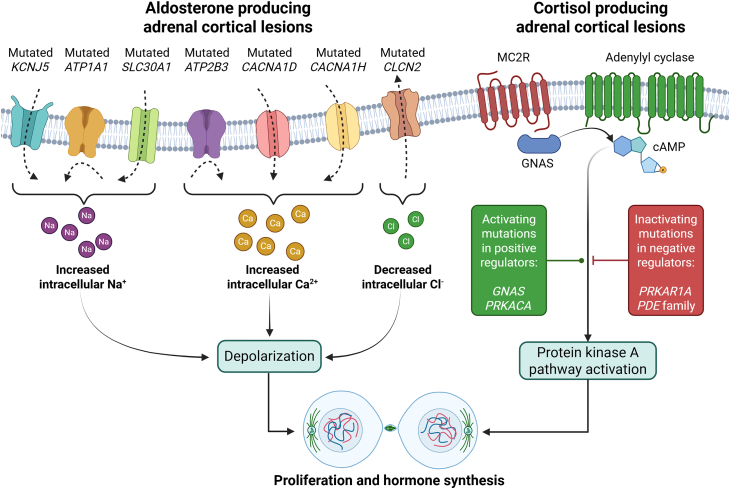
Main molecular aberrations in benign aldosterone- and cortisol-producing adrenocortical lesions In aldosterone-producing adenomas (left), somatic mutations in genes encoding membrane-bound ion channels play a central role in tumor development. These mutations either enhance the influx of cations into the cytoplasm - such as those in potassium, calcium, and zink channel genes (*KCNJ5*, *CACNA1D*, *CACNA1H,* and *SLC30A1*) - or impair the efflux of cations, as seen in mutations affecting ATPases (*ATP1A1* and *ATP2B3*). Moreover, rare mutations in the chloride channel gene *CLCN2* lead to an increased efflux of chloride ions. These mutations result in sustained cellular depolarization, which promotes cell proliferation and upregulates aldosterone synthase expression, driving autonomous hormone production. In cortisol-producing adenomas (right), genetic alterations affecting components of the cyclic AMP-driven protein kinase A (PKA) signaling cascade are commonly implicated. These include activating mutations in the G protein alpha subunit (*GNAS*) and the catalytic subunit of protein kinase A (*PRKACA*), as well as inactivating mutations in the regulatory subunit (*PRKAR1A*) or phosphodiesterase enzymes (*PDE* family genes). These disruptions lead to the constitutive activation of the pathway, resulting in increased cellular proliferation and cortisol biosynthesis. This figure was created using BioRender (https://biorender.com/).

Most APAs have been found to have somatic mutations in ion channels and ATPases, such as *KCNJ5*, *ATP1A1*, *ATP2B3*, *CACNA1D, CACNA1H, CLCN2,* and *SLC30A1,* as well as in the β-catenin (*CTNNB1*) gene (Figure 1). These ion channel and ATPase mutations cause dysregulated aldosterone synthesis by interfering with calcium signaling or other ion homeostasis.^25^^,^^26^^,^^27^ *KCNJ5* mutations are the most prevalent, particularly among younger patients and women, and have been associated with larger adenomas.^27^^,^^28^^,^^29^ Interestingly, there exists additional genotype-phenotype correlations with mild PA disproportionally driven by *CACNA1D*, *CACNA1H*, *ATP2B3*, *SLC30A1*, and *CADM1* mutations, moderate disease involves *ATP1A1*, *CLCN2*, and *CTNNB1* mutations, while severe PA is predominantly linked to *KCNJ5* and, less commonly, *ATP1A1* mutations.^30^^,^^31^^,^^32^ Gender and racial differences, such as higher *KCNJ5* mutation prevalence in East Asian women or *CACNA1D* in patients of African descent, also influence mutation distribution.^32^^,^^33^^,^^34^ Interestingly, mutations in the cell adhesion gene *CADM1* have also been implicated in PA, in which aberrant gap junction activity may impair cell-to-cell crosstalk, which in turn promotes proliferation and aldosterone synthase expression.^35^ In contrast, molecular dysregulation, such as changes in the expression of the gene encoding aldosterone synthase, *CYP11B2*, is increasingly recognized as the cause of APDH, which frequently lacks somatic mutations.^34^^,^^36^ Further illustrating the genetic heterogeneity of the disease, germline mutations have also been linked to familial forms of PA, including the *CYP11B1* and *CYP11B2* gene conversion (FH-I); *CLCN2* (FH-II); *KCNJ5* (FH-II); and *CACNA1H* (FH-IV).^32^^,^^34^^,^^37^

Beyond genetic mutations, epigenetic regulation has emerged as an important contributor to PA pathogenesis.^38^^,^^39^^,^^40^ Aberrant DNA methylation and microRNA dysregulation influence the expression of the aldosterone synthase gene (*CYP11B2*), resulting in autonomous aldosterone production.^34^^,^^36^^,^^41^^,^^42^ Overall, it appears that epigenetic mechanisms play a particularly important role in APDH, as the absence of discrete adenomas points to a more diffuse, molecularly mediated adrenal dysfunction. By identifying biomarkers and pathways that differentiate APAs from APDH and correlate with clinical phenotypes and outcomes, transcriptomic and proteomic analyses have further advanced our understanding of PA.^43^^,^^44^ Because they enable more precise therapeutic approaches and better diagnostic stratification, these molecular findings have important clinical ramifications. Preoperative prediction of *KCNJ5* mutation status, for instance, may help forecast surgical outcomes, enabling better patient selection for adrenalectomy, although this approach is not yet widely employed in clinical routine and currently relies on postoperative confirmation in most cases.

Despite these advancements, molecular insights have yet to be fully integrated into routine clinical practice, and many cases remain undiagnosed or undertreated. A deeper understanding of the genetic, epigenetic, and molecular mechanisms of PA is crucial for developing precision medicine approaches, including function-preserving adrenalectomy, particularly in patients with bilateral disease.^45^^,^^46^ Machine learning approaches are already delivering promising results in identifying patients with PA that may benefit from surgery.^47^ Recent advancements in diagnostic pathology have underscored the value of *CYP11B2* immunohistochemistry (IHC) in differentiating classic from non-classic histological patterns in aldosterone-producing lesions. The HISTALDO algorithm, now endorsed by the 2022 WHO Classification of Endocrine Tumors, incorporates *CYP11B2* staining into routine diagnostic workflows to classify aldosterone-producing adenomas (APAs) and related entities.^23^^,^^48^ Classic histology refers to solitary, benign *CYP11B2*-positive tumors that are almost always cured by unilateral adrenalectomy. In contrast, non-classic histology includes multiple *CYP11B2*-positive nodules (MAPN/MAPMN) or diffuse hyperplasia, which are linked with an increased risk of biochemically persistent or recurrent disease due to aldosterone hypersecretion from the contralateral adrenal gland.^48^^,^^49^ By improving diagnostic accuracy and facilitating more accurate stratification, this functional IHC-based method supports personalized clinical management.^23^^,^^48^ As a postoperative tool, HISTALDO informs prognostication and guides decisions regarding contralateral management, but it does not aid in initial surgical planning.

## Molecular pathology of cortisol-producing lesions

Cortisol-producing adrenal lesions, causing ACTH-independent hypercortisolism, may include subclinical forms such as MACS and overt adrenal Cushing syndrome. Pathologically, the benign lesions causing cortisol hypersecretion are classified as cortisol-producing adenoma (CPA), bilateral macronodular adrenocortical disease (BMAD, previously primary bilateral macronodular adrenal hyperplasia - PBMAH: multiple nodules size >1 cm), and bilateral micronodular adrenocortical disease (BMiNAD; multiple nodules size <1 cm).^23^ Autonomous cortisol production is the common feature of these disorders, which can result in hypercortisolism-related outcomes such as cardiovascular disease, osteoporosis, and metabolic syndrome. Recent advances in molecular genetics and genomics have shed light on the underlying mechanisms and their clinical significance, significantly expanding our understanding of the pathophysiology of lesions that produce cortisol. A key factor in the development of CPAs is somatic mutations in the cAMP/PKA signaling pathway. Over 35% of adenomas that produce cortisol have mutations in the *PRKACA* gene, which codes for the catalytic subunit of protein kinase A (PKA).^50^^,^^51^ These mutations result in the constitutive activation of the cAMP pathway, driving cortisol overproduction independent of ACTH stimulation. Additionally, *GNAS* mutations have been identified in a smaller subset of adrenal lesions, leading to the constitutive activation of G-protein coupled receptors^52^^,^^53^ (Figure 1). MACS-associated adenomas differ genetically from CPAs causing overt Cushing syndrome. They lack *PRKACA* mutations, whereas somatic mutations in the β-catenin (*CTNNB1*) and *GNAS* genes occur in about 23–65% and 5–25% of cases, respectively.^53^^,^^54^^,^^55^^,^^56^^,^^57^ Mutations in the β-catenin (*CTNNB1*) gene cause dysregulated Wnt/β-catenin signaling, promoting cell proliferation and modest cortisol hypersecretion.

Beyond somatic mutations, germline defects in genes such as *PRKAR1A*, *PRKACA*, *ARMC5, KDM1A, FH, APC,* and *MEN1* have been implicated in familial forms of adrenal cortical disorders and genetic syndromes associated with cortisol excess.^58^^,^^59^^,^^60^ In particular, *PRKAR1A* mutations are the cause of primary pigmented nodular adrenocortical disease (PPNAD) and the associated Carney complex (CNC).^61^ Germline defects in the related genes, *PRKACA*, *PRKACB*, *PRKAR1B, PDE8B,* and *PDE11A* were also found to rarely lead to inherited adrenal pathology associated with hypercortisolism.^62^^,^^63^^,^^64^ *ARMC5* and *KDM1A* mutations are linked to BMAD, a condition characterized by nodular adrenal enlargement and cortisol overproduction.^65^^,^^66^ *KDM1A* mutations in particular lead to a rare form of BMAD associated with food-dependent Cushing syndrome, a subtype of AHC, involving meal-stimulated cortisol release via aberrant GIP receptor (*GIPR*) signaling in adenomas.^66^^,^^67^ Germline variants in the *FH, APC,* and *MEN1* genes have been implicated in syndromic cases of BMAD, in the context of hereditary leiomyomatosis-kidney cancer syndrome, familial adenomatous polyposis, and multiple endocrine neoplasia type 1, respectively.^60^ These findings emphasize the importance of genetic screening in patients with bilateral adrenal disease and a strong familial predisposition.

It has also been demonstrated that epigenetic processes, such as abnormal DNA methylation and histone modifications, play a role in the dysregulation of important steroidogenic genes, including *CYP11B1*, which encodes the enzyme responsible for cortisol biosynthesis.^68^ Recent studies have demonstrated that microRNA dysregulation influences the expression of steroidogenic enzymes, which in turn affects the production of cortisol.^69^^,^^70^ Transcriptomic and proteomic analyses of cortisol-producing lesions have provided important information on the molecular pathways that drive hypercortisolism. Specific biomarkers have been identified by these studies and molecular signatures that distinguish CPAs from nonfunctioning adrenal tumors, enabling improved diagnostic precision and risk stratification.^57^^,^^71^ Despite these advancements, there is still a lack of integration of molecular findings into clinical practice, especially when it comes to MACS, where making decisions about diagnosis and treatment (surveillance versus surgery) can be difficult.

## Function-preserving adrenalectomy

The interest in function-preserving (partial) adrenalectomy has steadily increased over the past 2 decades. It has been well established in both animal models and humans that preserving approximately 30% of one adrenal gland preserves eucortisolism and does not lead to adrenal insufficiency.^19^^,^^72^ The key to successful partial adrenalectomy is to preserve sufficient normal adrenal cortex tissue (again, at least 30% of one adrenal gland; or 15% of both glands), and its blood supply.^6^^,^^19^ Function-preserving (partial) adrenalectomy is particularly attractive in patients with aldosterone- and cortisol-producing adrenal lesions since they are almost always benign, tend to be smaller, and there is a high prevalence of bilateral disease.^6^^,^^23^ It should be noted that function-preserving adrenalectomy should be used judiciously, especially in patients with a preoperative indeterminate or atypical imaging phenotype, since such tumors are associated with a higher risk of malignancy.^6^ Thus, such operations may be offered to patients thoughtfully and in a tailored fashion to the underlying pathology as outlined.^6^ The mini back scope adrenalectomy (MBSA, also known as posterior retroperitoneoscopic adrenalectomy; PRA) is particularly well suited for function-preserving (partial) adrenalectomy compared to other techniques, such as transabdominal laparoscopic and robotic adrenalectomy approaches.^6^^,^^19^ Unfortunately, there is a dearth of true high-volume adrenal surgeons, performing at least 100 adrenal operations per year, both in the United States and worldwide, and very few specialized endocrine surgery hospitals performing more than 500 adrenal operations per year.^6^ However, the use of adrenalectomy has increased in the United States,^73^ and is likely to continue to do so given that currently only a fraction of patients with potentially curative adrenal endocrinopathies (mainly aldosterone- and cortisol-producing adrenal lesions) are diagnosed and referred for adrenal surgery.^6^ Thus, it is likely that the enhanced expertise in adrenal surgery will rapidly increase in the coming years and expand the surgical treatment of aldosterone- and cortisol-producing adrenal lesions.

## Individualized surgery in primary aldosteronism

**Figure 2:**
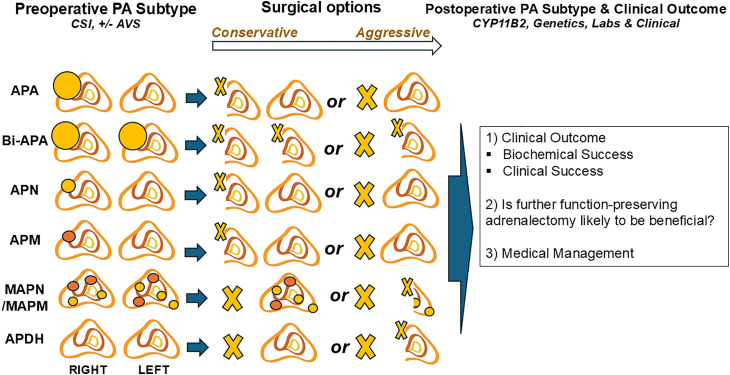
Individualized surgical approaches in primary aldosteronism The preoperative primary aldosteronism (PA) subtype is predicted based on cross-sectional imaging (CSI; typically, CT or MRI scans), with or without adrenal vein sampling (AVS). Following this, an individualized approach is used to decide whether surgical or medical management is indicated. For surgical candidates, the preoperative subtypes of benign PA include uni- and bilateral aldosterone-producing adenoma (APA and Bi-APA, respectively; size >1 cm), aldosterone-producing nodule (APN; size <1 cm), and aldosterone-producing micronodule (APM; size <1 cm, previously termed aldosterone-producing cell clusters). Both APNs and APMs may occur as multiple lesions (MAPN/MAPM), and like aldosterone-producing diffuse hyperplasia (APDH), are bilateral. The surgical options for each PA subtype are depicted (X indicates resected tissue) and range from conservative to aggressive and may include unilateral-partial, unilateral-total, bilateral-partial, and subtotal (total resection of one adrenal gland, and partial resection of the contralateral gland) adrenalectomy. Eucortisolism is accomplished by leaving a healthy adrenal remnant, *in situ*, the size of at least 30% of a normal adrenal gland. It is often preferred to perform surgery unilaterally (initial conservative approach), followed by a determination of the postoperative PA subtype, which is influenced by the pathological diagnosis, including *CYP11B2* IHC staining, molecular genetic information, biochemical and clinical outcomes according to established primary aldosteronism surgical outcome (PASO) scores. Most patients will achieve a total biochemical cure of PA after the initial operation. However, in patients with persistent PA, further function-preserving adrenalectomy is likely to be beneficial.

The only curative therapy for PA is the surgical resection of the aldosterone-producing lesion(s) via adrenalectomy, and the Endocrine Society recently published its updated guidelines for PA diagnosis and management.^10^ Once the diagnosis of PA has been established, preoperative cross-sectional imaging (CSI), typically with CT or MRI, is performed for PA subtype classification, surgical planning, and determination of whether adrenal vein sampling (AVS) is needed (Figure 2). Functional imaging, using PET-based technology, may be a promising adjunct in preoperative PA subtype classification, but is not yet widely available.^74^^,^^75^ The role of AVS in PA subtype classification remains controversial and evolving, where some centers use it routinely and some selectively.^5^^,^^76^ The major disadvantage with AVS is that it is an invasive procedure, technically challenging, and tends to be frequently performed by low-volume interventional radiologists, demonstrating very low success rates.^76^ Thus, numerous patients lack access to AVS or have failed AVS, which leads to denial of putative curative adrenalectomy.^76^^,^^77^ In general, we propose an individualized approach to AVS which factors in the patient’s geographic location, demographics, local expertise of AVS, the biochemical, clinical, and radiological findings, as well as the patient’s desire for uni- and bilateral function-preserving (partial) adrenalectomy.^6^ Some groups use segmental AVS to precisely map the intra-adrenal locations of the aldosterone-producing tumors, which enables partial adrenalectomy, although additional studies are warranted to compare its results versus conventional AVS.^78^^,^^79^ While AVS remains the gold standard for lateralization in PA, its limitations have spurred interest in functional imaging alternatives. However, PET imaging’s limited availability poses challenges, as well. Thus, further studies are needed to examine AVS and functional PET imaging in various subtypes of PA, and their roles in improving medical and surgical outcomes.^80^^,^^81^ Furthermore, an international consensus grading system classifies PA into mild, moderate, and severe categories, which may help guide the indication for AVS and surgical intervention, where moderate and severe cases are likely to benefit the most.^82^ In resource-limited settings, relying on conventional imaging alone combined with demographics (typically younger patients) and biochemical severity could be a strategy to avoid denying patients potentially curative surgery.

If total unilateral adrenalectomy is the only surgical option available, the management of primary aldosteronism (PA) relies more heavily on preoperative tools such as adrenal vein sampling (AVS) to ensure the correct side is targeted. However, with the increased availability of function-preserving adrenalectomy, the role of AVS is less critical since adrenal insufficiency can be avoided even in patients with bilateral PA. It is not controversial that unilateral adrenalectomy for unilateral PA has better outcomes, both clinically and biochemically, than unilateral adrenalectomy for bilateral PA.^7^^,^^8^ Similarly, it is uncontroversial that adrenalectomy for unilateral PA is superior to non-operative, pharmacological therapy and should be offered to patients whenever feasible.^5^^,^^83^ The role of adrenalectomy in bilateral PA is of great interest, and somewhat controversial. However, there is increasing evidence that unilateral or bilateral (function-preserving) adrenalectomy, even in bilateral PA, may be superior to medical management, at least in selected patients.^84^ Similarly, unilateral total adrenalectomy alone, in 24 patients with bilateral PA, resolved hypokalemia in 76% and improved blood pressure control in 65% of patients.^85^ It should be noted that these patients with bilateral PA (defined by AVS) undergoing surgery typically had more severe disease with overt biochemical abnormalities, frequent hypokalemia, and poorly controlled blood pressure despite escalating pharmacotherapy. Thus, the results should not be extrapolated to assume that adrenalectomy is superior to the medical treatment of all cases with bilateral PA, since such comparative studies have not been performed. In general, bilateral PA tends to be milder, with better response to medical therapy.^82^ Also, it should be noted that studies of function-preserving adrenalectomy may contain selection bias toward younger patients with more severe disease, potentially inflating success rates. For benign, non-familial PA, there is never an indication to render a patient permanently adrenally insufficient (i.e., performing a total bilateral adrenalectomy), although this may be necessary in rare cases of inherited, severe bilateral PA.^6^^,^^30^ Thus, preoperative knowledge of the causative germline mutations (e.g., in the *CLCN2*, *KCNJ5*, and *CACNA1H* genes) provides precision guidance for either function-preserving or total bilateral adrenalectomy.^32^^,^^34^^,^^37^

Based on the preoperative PA subtype classification using CSI with or without AVS, the surgical options range from conservative (more adrenal cell mass remains, *in situ*) to aggressive (less adrenal cell mass remains, *in situ*; Figure 2). The surgical option for unilateral APA would be unilateral function-preserving (partial, or enucleation; *conservative*) adrenalectomy or total unilateral adrenalectomy (*aggressive*). Similar options exist for the other PA subtypes as outlined in Figure 2. It has not been well studied whether performing bilateral (function-preserving) adrenalectomy simultaneously or in a staged fashion is superior. However, at our center, we typically prefer to perform these operations for PA in a staged fashion since initial unilateral adrenalectomy allows for postoperative subtype classification. Additionally, contralateral function-preserving (partial) adrenalectomy would then be performed based on the postoperative PA subtype classification, typically at least 6 weeks after the index operation. We believe this approach minimizes the risk for adrenal insufficiency and avoids unnecessary surgery in those patients with an excellent response to initial unilateral adrenalectomy.^6^ There exists a genotype-phenotype correlation in PA, where patients with e.g., *KCNJ5* mutations are associated with more pronounced PA, young age, female gender, larger tumors, and excellent surgical outcomes.^23^^,^^86^^,^^87^ Additionally, hybrid steroid profiling, which analyzes multisteroid metabolic patterns, shows promise for diagnosing and genotype subtyping of PA and may be a noninvasive indicator for predicting, e.g., *KCNJ5* mutations preoperatively, potentially aiding in surgical planning and patient stratification.^88^^,^^89^ Further studies correlating molecular pathology with postoperative outcomes, including biochemical cure, persistence, and recurrence rates as well as clinical outcomes such as improvements in hypokalemia, hypertension, and cardiovascular morbidity and mortality, are warranted. A thorough postoperative subtype classification is essential to assess clinical outcomes and determine whether further function-preserving adrenal resection is warranted or if medical management alone is preferable (Figure 2). No prospective trials yet demonstrate superiority over clinical criteria, and cost-effectiveness analyses are sparse. We recommend randomized studies comparing molecular-guided versus standard planning, with an emphasis on assessing biochemical cure and quality-of-life metrics.

## Personalized adrenalectomy in hypercortisolism

**Figure 3:**
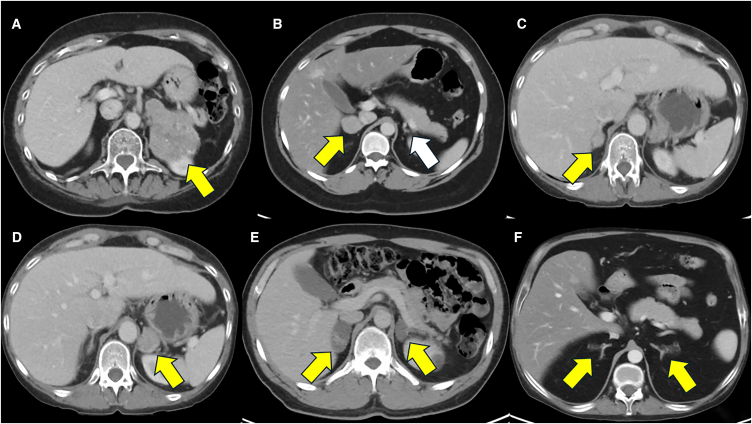
Representative CT scan images from patients with various subtypes of cortisol-producing adrenal lesions causing ACTH-independent Cushing’s syndrome (A) A 70-year-old woman with a large left adrenocortical carcinoma (ACC; yellow arrow), causing severe, overt adrenal Cushing’s syndrome. (B) A 25-year-old woman with a right adrenocortical adenoma (yellow arrow), causing overt adrenal Cushing syndrome (CPA), with a suppressed, atrophic right adrenal gland (white arrow). (C and D) A 43-year-old woman with bilateral CPAs (yellow arrows), causing overt adrenal Cushing syndrome. (E) A 49-year-old man with a bilateral macronodular adrenocortical disease (BMAD) (yellow arrows), causing subclinical Cushing syndrome (mild autonomous cortisol secretion; MACS). (F) A 31-year-old man with a bilateral micronodular adrenocortical disease (BMiNAD) (yellow arrows), causing severe, overt adrenal Cushing syndrome, in the setting of primary pigmented nodular adrenocortical disease (PPNAD). All CT scan images depicted are in venous phase (60 s following contrast injection), according to a modified adrenal protocol of the Carling Adrenal Center and the Hospital for Endocrine Surgery.

**Figure 4:**
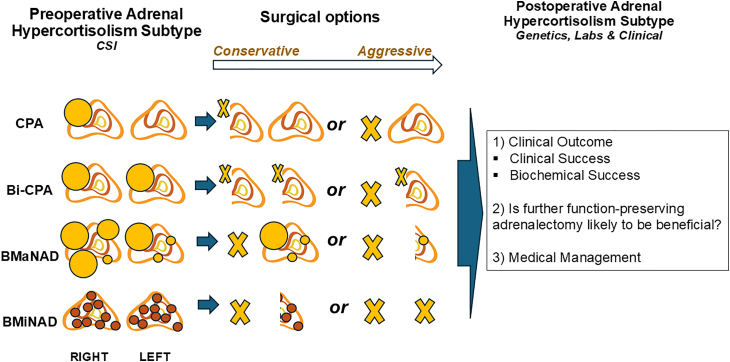
Personalized adrenalectomy in cortisol-producing adrenal lesions The preoperative subtype of adrenal hypercortisolism is predicted based on cross-sectional imaging (CSI; typically, CT or MRI scans) and may be overt (severe ACTH-independent Cushing syndrome) or subclinical (also known as mild autonomous cortisol secretion: MACS). Following this, a personalized approach is used to decide whether surgical, medical or surveillance management is indicated. There are 4 broad preoperative subtypes of benign adrenal hypercortisolism: uni- and bilateral cortisol-producing adenoma (CPA and Bi-CPA, respectively), bilateral macronodular adrenocortical disease (BMAD: multiple nodules size >1 cm), and bilateral micronodular adrenocortical disease (BMiNAD; multiple nodules size <1 cm, often in the setting of primary pigmented nodular adrenocortical disease; PPNAD). The surgical options for each adrenal hypercortisolism subtype are depicted (X indicates resected tissue) and range from conservative to aggressive and may include unilateral-partial, unilateral-total, bilateral-partial, and subtotal (total resection of one adrenal gland, and partial resection of the contralateral gland) and bilateral total adrenalectomy. Eucortisolism is most likely accomplished by leaving a healthy adrenal remnant, *in situ*, the size of at least 30% of a normal adrenal gland. However, due to the suppression of the cortisol secretion of the normal adjacent adrenocortical cells, a transient period of adrenal insufficiency is always a possibility even when an adequate remnant of adrenal tissue is left *in situ*. Thus, it is often preferred to perform surgery unilaterally (initial conservative approach), followed by a determination of the postoperative adrenal hypercortisolism subtype, which is influenced by the pathological diagnosis, molecular genetic information, and clinical outcomes. Function-preserving adrenalectomy should be avoided in patients with malignancy or suspicion of malignancy based on preoperative imaging.

ACTH-independent hypercortisolism and adrenal Cushing syndrome may be subclinical (*aka* MACS) or overt and are due to uni-or bilateral cortisol-producing lesions^9^^,^^90^ (Figure 3). Similar to PA, adrenalectomy is the only curative treatment, whether the clinical presentation is subclinical or overt. There exists no controversy that adrenalectomy should be offered to all patients with overt, severe adrenal Cushing syndrome, whenever feasible. Given that subclinical Cushing syndrome (MACS) is associated with cardiovascular morbidity, frailty, fragility fractures, decreased quality of life, and increased mortality, the use of adrenalectomy to reverse these effects of hypercortisolism is becoming increasingly attractive for patients and physicians alike.^4^^,^^17^ In fact, a recent randomized controlled trial recently demonstrated the superiority of adrenalectomy over medical management in reversing hypertension in patients with MACS.^91^ With the increased availability of function-preserving adrenalectomy, there are enhanced options for removing the cortisol-producing cells/tumor (whether uni-or bilateral), while preserving normal (or mostly normal) adjacent adrenal tissue, avoiding adrenal insufficiency. Similar to resecting PA adrenal lesions, the surgical options range from conservative (more adrenal cell mass remains, *in situ*) to aggressive (less adrenal cell mass remains, *in situ*; Figure 4).^6^^,^^19^ One study recently compared patients with unilateral adrenal Cushing syndrome (overt, *n* = 124 and subclinical, *n* = 17) treated with either partial or total unilateral adrenalectomy, demonstrating the shorter duration of corticosteroid therapy, and fewer patients requiring such therapy >2 years (4 vs. 25%).^20^ This suggests that even in patients undergoing solely unilateral adrenal surgery, preserving adrenal cell mass is beneficial in the setting of adrenal hypercortisolism.

For patients with bilateral lesions, such as BMAD and BMiNAD with frequent germline *ARMC5* and *PRKAR1A* mutations, there are three different operative strategies: bilateral total adrenalectomy, unilateral adrenalectomy guided by the size of the glands or result of AVS, and function-preserving (partial) bilateral adrenalectomy.^92^ Understanding a patient’s specific genotype frequently informs surgical decisions in bilateral lesions, as the remaining adrenal tissue is pathophysiologically not entirely normal. A bilateral function-preserving adrenalectomy approach strikes a balance, aiming to achieve biochemical resolution AHC while minimizing the need for glucocorticoid replacement therapy. Various function-preserving (partial) adrenalectomy approaches were used in two studies of patients with bilateral AHC, demonstrating overall cure rates of 92–98%, with low rates of both recurrence and long-term need for steroid hormone replacement.^93^^,^^94^ We do not recommend adrenal vein sampling (AVS) in this context, though some clinicians use it to identify the more affected side in patients with bilateral cortisol-producing adrenal lesions, particularly when total unilateral adrenalectomy is the only surgical option available.^90^^,^^95^^,^^96^ Since excess cortisol is still produced by the contralateral adrenal lesion(s), we favor bilateral function-preserving adrenalectomy. Similar to patients with PA, performing the operation in a staged fashion is often preferable to allow for the unilateral remnant to recover, typically for at least 6 weeks, prior to contralateral function-preserving (partial) adrenalectomy. In addition, some patients may have an excellent biochemical and clinical response to unilateral adrenalectomy alone, and thus contralateral function-preserving (partial) adrenalectomy can be delayed or even occasionally avoided completely. Furthermore, this approach reduces the risk of long-term adrenal insufficiency and the need for even transient glucocorticoid replacement therapy.^6^^,^^20^

A comprehensive postoperative subtype classification is necessary to evaluate the clinical outcome, establish whether additional function-preserving adrenal resection is likely to be beneficial, or if the patients should solely be managed medically (Figure 4). Patients are subject to a cosyntropin stimulation test at 4 a.m. the morning following adrenalectomy.^97^ The evaluation of cortisol at baseline and at 30- and 60-min following administration, respectively, leads to the improved identification of patients at risk for adrenal insufficiency, and avoids unnecessary glucocorticoid overtreatment in numerous patients. Conversely, it helps identify patients (mainly BMAD) with persistent hypercortisolemia after unilateral adrenalectomy, who are likely to benefit from additional function-preserving resection of the contralateral adrenal gland. In general, we prefer function-preserving adrenalectomy approaches for most patients with benign, cortisol-producing lesions, whether uni- or bilateral. Although not well studied, bilateral total adrenalectomy, rendering the patient adrenally insufficient, may be preferable in patients with a known pathogenic germline mutation (e.g., in the *PRKAR1A*, *ARMC5, KDM1A, PRKACA*, *PRKACB*, *PRKAR1B, PDE8B,* and *PDE11A* genes) causing bilateral ACTH-independent Cushing syndrome, especially those with severe hypercortisolism with overt Cushing syndrome.^92^^,^^98^^,^^99^ However, occasional patients with bilateral micronodular adrenocortical disease (BMiNAD), in the setting of primary pigmented nodular adrenocortical disease (PPNAD), have been managed with unilateral adrenalectomy.^100^ Similar to PA, additional studies correlating AHC molecular pathology with biochemical cure, persistence, and recurrence rates, as well as clinical outcomes such as improvements in comorbidity profiles as well as mortality rates are warranted.

## Conclusion and future perspectives

Recent advances in understanding the molecular etiology of PA and AHC have illuminated critical genetic and epigenetic drivers. Somatic mutations in ion channel genes, such as *KCNJ5*, *CACNA1D*, and *ATP1A1*, disrupt calcium signaling in PA, promoting autonomous aldosterone production, while cAMP/PKA pathway mutations, notably *PRKACA*, drive cortisol overproduction in AHC.^25^^,^^26^^,^^27^^,^^50^^,^^51^ Epigenetic mechanisms, including DNA methylation and microRNA dysregulation, further shape non-classic phenotypes by modulating steroidogenesis and hormone production.^70^^,^^101^ A cross-ancestry genome-wide association study revealed genetic heterogeneity in PA, highlighting the need for population-specific diagnostic tools.^102^ Longitudinal studies linking epigenetic profiles to clinical outcomes are crucial to translating these findings into targeted therapies that address underlying molecular defects and pave the way for precision medicine. Some key challenges are to improve case detection, diagnostic approaches, integrate lessons from molecular pathology, and enhance access to high-volume centers for adrenal surgery.

Diagnostic innovations are poised to enhance the management of PA and AHC. Functional imaging modalities, such as 11C-metomidate and 68Ga-pentixafor PET/CT, offer non-invasive alternatives to adrenal vein sampling (AVS) for PA subtype classification, demonstrating comparable accuracy in identifying unilateral disease and detecting dominant nodules in bilateral cases.^103^^,^^104^ Notably, molecular pathology, such as *KCNJ5* mutations in PA, correlates with PET positivity, underscoring an important research field to refine diagnostic accuracy and guide surgical planning.^75^^,^^105^ Circulating microRNAs show potential as biomarkers to differentiate PA and AHC from other forms of hypertension, facilitating earlier detection.^101^ Machine learning (ML) approaches, integrating biochemical, imaging, and genetic data, have shown promise in identifying surgical candidates and predicting postoperative outcomes in PA, potentially minimizing unnecessary interventions.^106^ Comparative trials pitting ML-guided diagnostics against traditional methods are needed to validate their utility and reshape preoperative workflows, ultimately improving diagnostic precision and patient selection for surgery. However, challenges include dataset biases, lack of external validation, and integration into clinical workflows. Prospective studies are needed to confirm applicability, particularly in resource-limited settings with real-world barriers such as cost and accessibility.

Pharmacological management remains essential for patients unsuitable for surgery or as a complement to surgical intervention. For patients with bilateral PA unsuitable for surgery (e.g., due to comorbidities), mineralocorticoid receptor antagonists (MRAs) such as spironolactone or eplerenone remain first-line therapy, effectively controlling hypertension and hypokalemia. However, long-term MRA use carries risks such as lack of libido, gynecomastia, or hyperkalemia, underscoring the value of surgical options such as partial adrenalectomy in select cases to achieve a cure while preserving function.^11^^,^^12^ Novel aldosterone synthase inhibitors (ASIs), such as Baxdrostat, and second-generation ASIs selectively reduce aldosterone levels in PA, offering a viable option for patients intolerant to mineralocorticoid receptor antagonists or those with bilateral disease, changing treatment paradigms.^107^^,^^108^ Phase 3 trials are needed to confirm long-term safety and efficacy compared to surgical approaches.^109^^,^^110^

For AHC, pharmacological therapies provide interim solutions for patients with comorbidities such as morbid obesity or severe diabetes, controlling severe hypercortisolemia before surgery.^111^ Preoperative pharmacological optimization may reduce postoperative complications, such as glucocorticoid withdrawal syndrome, but randomized trials are required to establish optimal regimens and quantify their benefits, particularly in enhancing surgical outcomes.

Adrenal surgery has been revolutionized by minimally invasive techniques, notably MBSA, which has become the standard for managing benign adrenal tumors. MBSA enables function-preserving partial adrenalectomies, reducing morbidity, shortening hospital stays, and accelerating recovery while preserving sufficient adrenal tissue to maintain eucortisolism and avoid lifelong adrenal insufficiency.^6^^,^^22^ To address adrenal surgical capacity, expertise, and worldwide disparities, establishing high-volume adrenal centers, enhanced training, and collaboration via referral networks are critical to ensure equitable access to specialized care.^80^^,^^112^

Molecular profiling, identifying mutations such as *KCNJ5* in PA (associated with favorable surgical outcomes) or *PRKACA* in AHC, informs individualized surgical strategies that target hormone-producing lesions while sparing healthy tissue.^113^ The optimal approach to bilateral function-preserving adrenalectomy—whether simultaneous or staged—remains under investigation, with staged procedures, typically spaced six weeks apart, allowing remnant recovery and postoperative subtype confirmation to minimize hypocortisolism.^6^ Preliminary outcomes demonstrate high cure rates and low rates of adrenal insufficiency in bilateral disease after bilateral function-preserving adrenalectomy for both PA and AHC.^22^^,^^114^ Trials comparing surgical and pharmacological approaches are essential to evaluate cardiovascular outcomes, recurrence rates, and cost-effectiveness, ensuring that treatment strategies are tailored to individual patient needs. Furthermore, several multicenter randomized controlled trials comparing various adrenalectomy approaches (e.g., total unilateral versus function-preserving bilateral) are warranted for various subtypes of both PA and AHC, examining, e.g., biochemical cure, recurrence, and quality-of-life outcomes.

In conclusion, an integrated approach to PA and AHC, driven by a deeper understanding of their molecular and physiological mechanisms from laboratory research to surgical practice, is poised to enhance patient outcomes and broaden the indications for adrenalectomy as the morbidity of these endocrinopathies gains greater recognition.

## Acknowledgments

This research did not receive any specific grant from any funding agency in the public, commercial, or not-for-profit sector. Institutional support was provided by HCA Healthcare and/or an HCA Healthcare-affiliated entity. The views expressed do not necessarily represent those of HCA Healthcare or its affiliates.

## Declaration of interests

C.A.S. received grant funding from Pfizer and compensation from ELPEN Pharmaceuticals, Lundbeck, Sterotherapeutics, and Human Longevity Inc. C.A.S. also holds patents on *GPR101*, *PRKAR1A,* and *PDE11A* genes and their applications; FRF holds patents on the *GPR101* gene and its applications; T.C. and C.C.J. declare no competing interests.

## References

[1] Reincke M., Bancos I., Mulatero P., Scholl U.I., Stowasser M., Williams T.A. (2021). Diagnosis and treatment of primary aldosteronism. Lancet Diabetes Endocrinol..

[2] Reincke M., Fleseriu M. (2023). Cushing Syndrome: A Review. JAMA.

[3] Lacroix A., Feelders R.A., Stratakis C.A., Nieman L.K. (2015). Cushing's syndrome. Lancet.

[4] Starker L.F., Kunstman J.W., Carling T. (2014). Subclinical Cushing syndrome: a review. Surg. Clin. North Am..

[5] Funder J.W., Carey R.M., Mantero F., Murad M.H., Reincke M., Shibata H., Stowasser M., Young W.F. (2016). The Management of Primary Aldosteronism: Case Detection, Diagnosis, and Treatment: An Endocrine Society Clinical Practice Guideline. J. Clin. Endocrinol. Metab..

[6] Carling T., LaRue M. (2025). Improved and individualized approach to adrenal surgery. Endocr. Relat. Cancer.

[7] Meyer L.S., Handgriff L., Lim J.S., Udager A.M., Kinker I.S., Ladurner R., Wildgruber M., Knösel T., Bidlingmaier M., Rainey W.E. (2021). Single-Center Prospective Cohort Study on the Histopathology, Genotype, and Postsurgical Outcomes of Patients With Primary Aldosteronism. Hypertension.

[8] Williams T.A., Lenders J.W.M., Mulatero P., Burrello J., Rottenkolber M., Adolf C., Satoh F., Amar L., Quinkler M., Deinum J. (2017). Outcomes after adrenalectomy for unilateral primary aldosteronism: an international consensus on outcome measures and analysis of remission rates in an international cohort. Lancet Diabetes Endocrinol..

[9] Stratakis C.A. (2008). Cushing syndrome caused by adrenocortical tumors and hyperplasias (corticotropin- independent Cushing syndrome). Endocr. Dev..

[10] Adler G.K., Stowasser M., Correa R.R., Khan N., Kline G., McGowan M.J., Mulatero P., Murad M.H., Touyz R.M., Vaidya A. (2025). Primary Aldosteronism: An Endocrine Society Clinical Practice Guideline. J. Clin. Endocrinol. Metab..

[11] Rossi G.P., Rossi F.B., Guarnieri C., Rossitto G., Seccia T.M. (2024). Clinical Management of Primary Aldosteronism: An Update. Hypertension.

[12] Lechner B., Lechner K., Heinrich D., Adolf C., Holler F., Schneider H., Beuschlein F., Reincke M. (2019). THERAPY OF ENDOCRINE DISEASE: Medical treatment of primary aldosteronism. Eur. J. Endocrinol..

[13] Fleseriu M., Auchus R.J., Bancos I., Biller B.M.K. (2025). Osilodrostat Treatment for Adrenal and Ectopic Cushing Syndrome: Integration of Clinical Studies With Case Presentations. J. Endocr. Soc..

[14] Chen S.Y., Chen J.Y., Huang W.C., Puar T.H.K., Chin Kek P., Chueh J.S., Lin Y.H., Wu V.C., Taipai Study Group (2022). Cardiovascular outcomes and all-cause mortality in primary aldosteronism after adrenalectomy or mineralocorticoid receptor antagonist treatment: a meta-analysis. Eur. J. Endocrinol..

[15] Farah M.H., Hegazi M., Firwana M., Abusalih M., Saadi S., Al-Kordi M., Elsheikh A., Wang Z., Hassett L., Bancos I., Murad M.H. (2025). A Systematic Review Supporting the Endocrine Society Clinical Practice Guideline on Management of Primary Aldosteronism. J. Clin. Endocrinol. Metab..

[16] Gkaniatsa E., Zverkova Sandström T., Rosengren A., Trimpou P., Olsson D.S., Lind M., Muth A., Johannsson G., Ragnarsson O. (2023). Mortality in Patients With Primary Aldosteronism: A Swedish Nationwide Study. Hypertension.

[17] Prete A., Bancos I. (2024). Mild autonomous cortisol secretion: pathophysiology, comorbidities and management approaches. Nat. Rev. Endocrinol..

[18] Clayton R.N. (2022). Cardiovascular complications of Cushings syndrome: Impact on morbidity and mortality. J. Neuroendocrinol..

[19] Alesina P.F., Knyazeva P., Hinrichs J., Walz M.K. (2022). Tailored Approach in Adrenal Surgery: Retroperitoneoscopic Partial Adrenalectomy. Front. Endocrinol..

[20] Alesina P.F., Kniazeva P., Pinto G., Pontin A., Walz M.K. (2024). Long-term outcome of retroperitoneoscopic partial versus total adrenalectomy in patients with Cushing's syndrome. World J. Surg..

[21] Carling T. (2010). Retroperitoneal endoscopic adrenalectomy is safe and effective (Br J Surg 2010; 97:1667-1672). Br. J. Surg..

[22] Knyazeva P., Buzanakov D., Alesina P.F., Walz M.K. (2025). Partial adrenalectomy by the posterior retroperitoneoscopic approach: A single institution series of 766 consecutive procedures. World J. Surg..

[23] Mete O., Erickson L.A., Juhlin C.C., de Krijger R.R., Sasano H., Volante M., Papotti M.G. (2022). Overview of the 2022 WHO Classification of Adrenal Cortical Tumors. Endocr. Pathol..

[24] Turcu A.F., Yang J., Vaidya A. (2022). Primary aldosteronism - a multidimensional syndrome. Nat. Rev. Endocrinol..

[25] Beuschlein F., Boulkroun S., Osswald A., Wieland T., Nielsen H.N., Lichtenauer U.D., Penton D., Schack V.R., Amar L., Fischer E. (2013). Somatic mutations in ATP1A1 and ATP2B3 lead to aldosterone-producing adenomas and secondary hypertension. Nat. Genet..

[26] Scholl U.I., Goh G., Stölting G., de Oliveira R.C., Choi M., Overton J.D., Fonseca A.L., Korah R., Starker L.F., Kunstman J.W. (2013). Somatic and germline CACNA1D calcium channel mutations in aldosterone-producing adenomas and primary aldosteronism. Nat. Genet..

[27] Williams T.A., Monticone S., Schack V.R., Stindl J., Burrello J., Buffolo F., Annaratone L., Castellano I., Beuschlein F., Reincke M. (2014). Somatic ATP1A1, ATP2B3, and KCNJ5 mutations in aldosterone-producing adenomas. Hypertension.

[28] Akerstrom T., Crona J., Delgado Verdugo A., Starker L.F., Cupisti K., Willenberg H.S., Knoefel W.T., Saeger W., Feller A., Ip J. (2012). Comprehensive re-sequencing of adrenal aldosterone producing lesions reveal three somatic mutations near the KCNJ5 potassium channel selectivity filter. PLoS One.

[29] Boulkroun S., Beuschlein F., Rossi G.P., Golib-Dzib J.F., Fischer E., Amar L., Mulatero P., Samson-Couterie B., Hahner S., Quinkler M. (2012). Prevalence, clinical, and molecular correlates of KCNJ5 mutations in primary aldosteronism. Hypertension.

[30] Choi M., Scholl U.I., Yue P., Björklund P., Zhao B., Nelson-Williams C., Ji W., Cho Y., Patel A., Men C.J. (2011). K+ channel mutations in adrenal aldosterone-producing adenomas and hereditary hypertension. Science.

[31] Ip J.C.Y., Pang T.C.Y., Pon C.K., Zhao J.T., Sywak M.S., Gill A.J., Soon P.S., Sidhu S.B. (2015). Mutations in KCNJ5 determines presentation and likelihood of cure in primary hyperaldosteronism. ANZ J. Surg..

[32] Scholl U.I. (2022). Genetics of Primary Aldosteronism. Hypertension.

[33] Nanba K., Omata K., Gomez-Sanchez C.E., Stratakis C.A., Demidowich A.P., Suzuki M., Thompson L.D.R., Cohen D.L., Luther J.M., Gellert L. (2019). Genetic Characteristics of Aldosterone-Producing Adenomas in Blacks. Hypertension.

[34] Zennaro M.C., Boulkroun S., Fernandes-Rosa F.L. (2020). Pathogenesis and treatment of primary aldosteronism. Nat. Rev. Endocrinol..

[35] Wu X., Azizan E.A.B., Goodchild E., Garg S., Hagiyama M., Cabrera C.P., Fernandes-Rosa F.L., Boulkroun S., Kuan J.L., Tiang Z. (2023). Somatic mutations of CADM1 in aldosterone-producing adenomas and gap junction-dependent regulation of aldosterone production. Nat. Genet..

[36] Nakamura Y., Yamazaki Y., Tezuka Y., Satoh F., Sasano H. (2016). Expression of CYP11B2 in Aldosterone-Producing Adrenocortical Adenoma: Regulatory Mechanisms and Clinical Significance. Tohoku J. Exp. Med..

[37] Pitsava G., Faucz F.R., Stratakis C.A., Hannah-Shmouni F. (2022). Update on the Genetics of Primary Aldosteronism and Aldosterone-Producing Adenomas. Curr. Cardiol. Rep..

[38] Murakami M., Yoshimoto T., Nakabayashi K., Tsuchiya K., Minami I., Bouchi R., Izumiyama H., Fujii Y., Abe K., Tayama C. (2015). Integration of transcriptome and methylome analysis of aldosterone-producing adenomas. Eur. J. Endocrinol..

[39] Armignacco R., Reel P.S., Reel S., Jouinot A., Septier A., Gaspar C., Perlemoine K., Larsen C.K., Bouys L., Braun L. (2022). Whole blood methylome-derived features to discriminate endocrine hypertension. Clin. Epigenet..

[40] Howard B., Wang Y., Xekouki P., Faucz F.R., Jain M., Zhang L., Meltzer P.G., Stratakis C.A., Kebebew E. (2014). Integrated analysis of genome-wide methylation and gene expression shows epigenetic regulation of CYP11B2 in aldosteronomas. J. Clin. Endocrinol. Metab..

[41] Zhang G., Zou X., Liu Q., Xie T., Huang R., Kang H., Lai C., Zhu J. (2018). MiR-193a-3p functions as a tumour suppressor in human aldosterone-producing adrenocortical adenoma by down-regulating CYP11B2. Int. J. Exp. Pathol..

[42] Nakano Y., Yoshimoto T., Watanabe R., Murakami M., Fukuda T., Saito K., Fujii Y., Akashi T., Tanaka T., Yamada T. (2019). miRNA299 involvement in CYP11B2 expression in aldosterone-producing adenoma. Eur. J. Endocrinol..

[43] Swierczynska M.M., Betz M.J., Colombi M., Dazert E., Jenö P., Moes S., Pfaff C., Glatz K., Reincke M., Beuschlein F. (2019). Proteomic Landscape of Aldosterone-Producing Adenoma. Hypertension.

[44] Zhou J., Lam B., Neogi S.G., Yeo G.S.H., Azizan E.A.B., Brown M.J. (2016). Transcriptome Pathway Analysis of Pathological and Physiological Aldosterone-Producing Human Tissues. Hypertension.

[45] Stratakis C.A. (2023). O tempora, o mores: The Age We Live In, Machine Learning, Hypertension, and Primary Aldosteronism. JACC Asia.

[46] Kaneko H., Umakoshi H., Ogata M., Wada N., Iwahashi N., Fukumoto T., Yokomoto-Umakoshi M., Nakano Y., Matsuda Y., Miyazawa T. (2021). Machine learning based models for prediction of subtype diagnosis of primary aldosteronism using blood test. Sci. Rep..

[47] Chen L.C., Huang W.C., Peng K.Y., Chen Y.Y., Li S.C., Syed Mohammed Nazri S.K., Lin Y.H., Lin L.Y., Lu T.M., Kim J.H. (2023). Identifying KCNJ5 Mutation in Aldosterone-Producing Adenoma Patients With Baseline Characteristics Using Machine Learning Technology. JACC Asia.

[48] Williams T.A., Gomez-Sanchez C.E., Rainey W.E., Giordano T.J., Lam A.K., Marker A., Mete O., Yamazaki Y., Zerbini M.C.N., Beuschlein F. (2021). International Histopathology Consensus for Unilateral Primary Aldosteronism. J. Clin. Endocrinol. Metab..

[49] Volpe C., Hamberger B., Zedenius J., Juhlin C.C. (2020). Impact of immunohistochemistry on the diagnosis and management of primary aldosteronism: An important tool for improved patient follow-up. Scand. J. Surg..

[50] Beuschlein F., Fassnacht M., Assié G., Calebiro D., Stratakis C.A., Osswald A., Ronchi C.L., Wieland T., Sbiera S., Faucz F.R. (2014). Constitutive activation of PKA catalytic subunit in adrenal Cushing's syndrome. N. Engl. J. Med..

[51] Cao Y., He M., Gao Z., Peng Y., Li Y., Li L., Zhou W., Li X., Zhong X., Lei Y. (2014). Activating hotspot L205R mutation in PRKACA and adrenal Cushing's syndrome. Science.

[52] Angelousi A., Fencl F., Faucz F.R., Malikova J., Sumnik Z., Lebl J., Stratakis C.A. (2015). McCune Albright syndrome and bilateral adrenal hyperplasia: the GNAS mutation may only be present in adrenal tissue. Hormones (Basel).

[53] Goh G., Scholl U.I., Healy J.M., Choi M., Prasad M.L., Nelson-Williams C., Kunstman J.W., Korah R., Suttorp A.C., Dietrich D. (2014). Recurrent activating mutation in PRKACA in cortisol-producing adrenal tumors. Nat. Genet..

[54] Tissier F., Cavard C., Groussin L., Perlemoine K., Fumey G., Hagneré A.M., René-Corail F., Jullian E., Gicquel C., Bertagna X. (2005). Mutations of beta-catenin in adrenocortical tumors: activation of the Wnt signaling pathway is a frequent event in both benign and malignant adrenocortical tumors. Cancer Res..

[55] Ronchi C.L., Di Dalmazi G., Faillot S., Sbiera S., Assié G., Weigand I., Calebiro D., Schwarzmayr T., Appenzeller S., Rubin B. (2016). Genetic Landscape of Sporadic Unilateral Adrenocortical Adenomas Without PRKACA p.Leu206Arg Mutation. J. Clin. Endocrinol. Metab..

[56] Pitsava G., Stratakis C.A. (2022). Genetic Alterations in Benign Adrenal Tumors. Biomedicines.

[57] Rege J., Hoxie J., Liu C.J., Cash M.N., Luther J.M., Gellert L., Turcu A.F., Else T., Giordano T.J., Udager A.M. (2022). Targeted Mutational Analysis of Cortisol-Producing Adenomas. J. Clin. Endocrinol. Metab..

[58] Assie G., Libe R., Espiard S., Rizk-Rabin M., Guimier A., Luscap W., Barreau O., Lefevre L., Sibony M., Guignat L. (2013). ARMC5 mutations in macronodular adrenal hyperplasia with Cushing's syndrome. N. Engl. J. Med..

[59] Gatta-Cherifi B., Chabre O., Murat A., Niccoli P., Cardot-Bauters C., Rohmer V., Young J., Delemer B., Du Boullay H., Verger M.F. (2012). Adrenal involvement in MEN1. Analysis of 715 cases from the Groupe d'etude des Tumeurs Endocrines database. Eur. J. Endocrinol..

[60] Vaduva P., Bertherat J. (2024). The molecular genetics of adrenal cushing. Hormones (Basel).

[61] Pitsava G., Stratakis C.A. (2022). Genetic Alterations in Benign Adrenal Tumors. Biomedicines.

[62] Lodish M.B., Yuan B., Levy I., Braunstein G.D., Lyssikatos C., Salpea P., Szarek E., Karageorgiadis A.S., Belyavskaya E., Raygada M. (2015). Germline PRKACA amplification causes variable phenotypes that may depend on the extent of the genomic defect: molecular mechanisms and clinical presentations. Eur. J. Endocrinol..

[63] Espiard S., Drougat L., Settas N., Haydar S., Bathon K., London E., Levy I., Faucz F.R., Calebiro D., Bertherat J. (2020). PRKACB variants in skeletal disease or adrenocortical hyperplasia: effects on protein kinase A. Endocr. Relat. Cancer.

[64] Drougat L., Settas N., Ronchi C.L., Bathon K., Calebiro D., Maria A.G., Haydar S., Voutetakis A., London E., Faucz F.R., Stratakis C.A. (2021). Genomic and sequence variants of protein kinase A regulatory subunit type 1β (PRKAR1B) in patients with adrenocortical disease and Cushing syndrome. Genet. Med..

[65] Espiard S., Drougat L., Libé R., Assié G., Perlemoine K., Guignat L., Barrande G., Brucker-Davis F., Doullay F., Lopez S. (2015). ARMC5 Mutations in a Large Cohort of Primary Macronodular Adrenal Hyperplasia: Clinical and Functional Consequences. J. Clin. Endocrinol. Metab..

[66] Bouys L., Vaduva P., Jouinot A., Violon F., Vaczlavik A., Barat M., Charchar H., Chasseloup F., Kamilaris C., Espiard S. (2025). KDM1A genetic alterations, a rare cause of primary bilateral macronodular adrenal hyperplasia, strongly associated with food-dependent Cushing's syndrome: results of its systematic germline screening in 301 index cases and genotype/phenotype correlation. Eur. J. Endocrinol..

[67] Lecoq A.L., Stratakis C.A., Viengchareun S., Chaligné R., Tosca L., Deméocq V., Hage M., Berthon A., Faucz F.R., Hanna P. (2017). Adrenal GIPR expression and chromosome 19q13 microduplications in GIP-dependent Cushing's syndrome. JCI Insight.

[68] Takeda Y., Demura M., Kometani M., Karashima S., Yoneda T., Takeda Y. (2023). Molecular and Epigenetic Control of Aldosterone Synthase, CYP11B2 and 11-Hydroxylase, CYP11B1. Int. J. Mol. Sci..

[69] Robertson S., Diver L.A., Alvarez-Madrazo S., Livie C., Ejaz A., Fraser R., Connell J.M., MacKenzie S.M., Davies E. (2017). Regulation of Corticosteroidogenic Genes by MicroRNAs. Int. J. Endocrinol..

[70] Vetrivel S., Zhang R., Engel M., Oßwald A., Watts D., Chen A., Wielockx B., Sbiera S., Reincke M., Riester A. (2022). Characterization of Adrenal miRNA-Based Dysregulations in Cushing's Syndrome. Int. J. Mol. Sci..

[71] Iwahashi N., Umakoshi H., Ogata M., Fukumoto T., Kaneko H., Terada E., Katsuhara S., Uchida N., Sasaki K., Yokomoto-Umakoshi M. (2022). Whole Transcriptome Profiling of Adrenocortical Tumors Using Formalin-Fixed Paraffin-Embedded Samples. Front. Endocrinol..

[72] Walz M.K., Peitgen K., Diesing D., Petersenn S., Janssen O.E., Philipp T., Metz K.A., Mann K., Schmid K.W., Neumann H.P.H. (2004). Partial versus total adrenalectomy by the posterior retroperitoneoscopic approach: early and long-term results of 325 consecutive procedures in primary adrenal neoplasias. World J. Surg..

[73] Saunders B.D., Wainess R.M., Dimick J.B., Upchurch G.R., Doherty G.M., Gauger P.G. (2004). Trends in utilization of adrenalectomy in the United States: have indications changed?. World J. Surg..

[74] Wu X., Senanayake R., Goodchild E., Bashari W.A., Salsbury J., Cabrera C.P., Argentesi G., O'Toole S.M., Matson M., Koo B. (2023). [(11)C]metomidate PET-CT versus adrenal vein sampling for diagnosing surgically curable primary aldosteronism: a prospective, within-patient trial. Nat. Med..

[75] Zhang X., Song Y., Jing Y., Hu J., Shen H., Zhang A., He W., Feng Z., Yang Y., Pang H. (2025). Comparison of Different Diagnostic Criteria of 68Ga-Pentixafor PET/CT for the Classification of Primary Aldosteronism. J. Clin. Endocrinol. Metab..

[76] Turcu A.F., Auchus R. (2021). Approach to the Patient with Primary Aldosteronism: Utility and Limitations of Adrenal Vein Sampling. J. Clin. Endocrinol. Metab..

[77] Buffolo F., Monticone S., Williams T.A., Rossato D., Burrello J., Tetti M., Veglio F., Mulatero P. (2017). Subtype Diagnosis of Primary Aldosteronism: Is Adrenal Vein Sampling Always Necessary?. Int. J. Mol. Sci..

[78] Kitamoto T., Kitamoto K.K., Omura M., Takiguchi T., Tsurutani Y., Kubo H., Yamazaki Y., Sasano H., Saito J., Nishikawa T. (2020). Precise Mapping of Intra-Adrenal Aldosterone Activities Provides a Novel Surgical Strategy for Primary Aldosteronism. Hypertension.

[79] Turcu A.F., Gomez-Sanchez C.E. (2020). Segmental Adrenal Vein Sampling in Patients With Primary Aldosteronism: Superlative or Superfluous?. Hypertension.

[80] Carling T., Faucz F.R. (2025). Primary aldosteronism: adrenalectomy could save more lives. Front. Endocrinol..

[81] Deinum J., Turcu A.F. (2025). Adrenal Vein Sampling in Primary Aldosteronism-Is The Gold Standard Losing Its Luster?. J. Clin. Endocrinol. Metab..

[82] Murakami M., Naruse M., Kobayashi H., Parasiliti-Caprino M., Bioletto F., Brüdgam D., Stüfchen I., Reincke M., St-Jean M., Kraljevic I. (2025). Expert Consensus on the Primary Aldosteronism Severity Classification and its strategic application in indicating adrenal venous sampling. Eur. J. Endocrinol..

[83] Mullen N., Curneen J., Donlon P.T., Prakash P., Bancos I., Gurnell M., Dennedy M.C. (2024). Treating Primary Aldosteronism-Induced Hypertension: Novel Approaches and Future Outlooks. Endocr. Rev..

[84] Williams T.A., Gong S., Tsurutani Y., Tezuka Y., Thuzar M., Burrello J., Wu V.C., Yamazaki Y., Mulatero P., Sasano H. (2022). Adrenal surgery for bilateral primary aldosteronism: an international retrospective cohort study. Lancet Diabetes Endocrinol..

[85] Szabo Yamashita T., Shariq O.A., Foster T.R., Lyden M.L., Dy B.M., Young W.F., Bancos I., McKenzie T.J. (2023). Unilateral Adrenalectomy for Primary Aldosteronism Due to Bilateral Adrenal Disease Can Result in Resolution of Hypokalemia and Amelioration of Hypertension. World J. Surg..

[86] Lenzini L., Rossitto G., Maiolino G., Letizia C., Funder J.W., Rossi G.P. (2015). A Meta-Analysis of Somatic KCNJ5 K(+) Channel Mutations In 1636 Patients With an Aldosterone-Producing Adenoma. J. Clin. Endocrinol. Metab..

[87] Scholl U.I., Healy J.M., Thiel A., Fonseca A.L., Brown T.C., Kunstman J.W., Horne M.J., Dietrich D., Riemer J., Kücükköylü S. (2015). Novel somatic mutations in primary hyperaldosteronism are related to the clinical, radiological and pathological phenotype. Clin. Endocrinol..

[88] Knuchel R., Erlic Z., Gruber S., Amar L., Larsen C.K., Gimenez-Roqueplo A.P., Mulatero P., Tetti M., Pecori A., Pamporaki C. (2024). Association of adrenal steroids with metabolomic profiles in patients with primary and endocrine hypertension. Front. Endocrinol..

[89] Murakami M., Rhayem Y., Kunzke T., Sun N., Feuchtinger A., Ludwig P., Strom T.M., Gomez-Sanchez C., Knösel T., Kirchner T. (2019). In situ metabolomics of aldosterone-producing adenomas. JCI Insight.

[90] Bouys L., Violon F., Louiset E., Sibony M., Lefebvre H., Bertherat J. (2024). Bilateral Adrenocortical Nodular Disease and Cushing's Syndrome. J. Clin. Endocrinol. Metab..

[91] Tabarin A., Espiard S., Deutschbein T., Amar L., Vezzossi D., Di Dalmazi G., Reznik Y., Young J., Desailloud R., Goichot B. (2025). Surgery for the treatment of arterial hypertension in patients with unilateral adrenal incidentalomas and mild autonomous cortisol secretion (CHIRACIC): a multicentre, open-label, superiority randomised controlled trial. Lancet Diabetes Endocrinol..

[92] Bertherat J., Bourdeau I., Bouys L., Chasseloup F., Kamenický P., Lacroix A. (2023). Clinical, Pathophysiologic, Genetic, and Therapeutic Progress in Primary Bilateral Macronodular Adrenal Hyperplasia. Endocr. Rev..

[93] He H.C., Dai J., Shen Z.J., Zhu Y., Sun F.K., Shao Y., Zhang R.M., Wang H.F., Rui W.B., Zhong S. (2012). Retroperitoneal adrenal-sparing surgery for the treatment of Cushing's syndrome caused by adrenocortical adenoma: 8-year experience with 87 patients. World J. Surg..

[94] Lowery A.J., Seeliger B., Alesina P.F., Walz M.K. (2017). Posterior retroperitoneoscopic adrenal surgery for clinical and subclinical Cushing's syndrome in patients with bilateral adrenal disease. Langenbecks Arch. Surg..

[95] Ueland G.Å., Methlie P., Jøssang D.E., Sagen J.V., Viste K., Thordarson H.B., Heie A., Grytaas M., Løvås K., Biermann M., Husebye E.S. (2018). Adrenal Venous Sampling for Assessment of Autonomous Cortisol Secretion. J. Clin. Endocrinol. Metab..

[96] Johnson P.C., Thompson S.M., Adamo D., Fleming C.J., Bancos I., McKenzie T.J., Cheville J., Young W.F., Andrews J.C. (2023). Adrenal venous sampling for lateralization of cortisol hypersecretion in patients with bilateral adrenal masses. Clin. Endocrinol..

[97] DeLozier O.M., Dream S.Y., Findling J.W., Carroll T.B., Evans D.B., Wang T.S. (2022). Selective Glucocorticoid Replacement Following Unilateral Adrenalectomy for Hypercortisolism and Primary Aldosteronism. J. Clin. Endocrinol. Metab..

[98] Powell A.C., Stratakis C.A., Patronas N.J., Steinberg S.M., Batista D., Alexander H.R., Pingpank J.F., Keil M., Bartlett D.L., Libutti S.K. (2008). Operative management of Cushing syndrome secondary to micronodular adrenal hyperplasia. Surgery.

[99] Cavalcante I.P., Berthon A., Fragoso M.C., Reincke M., Stratakis C.A., Ragazzon B., Bertherat J. (2022). Primary bilateral macronodular adrenal hyperplasia: definitely a genetic disease. Nat. Rev. Endocrinol..

[100] Vitellius G., Donadille B., Decoudier B., Leroux A., Deguelte S., Barraud S., Bertherat J., Delemer B. (2022). Unilateral or bilateral adrenalectomy in PPNAD: six cases from a single family followed up over 40 years. Endocrine.

[101] Reel S., Reel P.S., Van Kralingen J., Larsen C.K., Robertson S., MacKenzie S.M., Riddell A., McClure J.D., Lamprou S., Connell J.M.C. (2025). Identification of hypertension subtypes using microRNA profiles and machine learning. Eur. J. Endocrinol..

[102] Naito T., Inoue K., Sonehara K., Baba R., Kodama T., Otagaki Y., Okada A., Itcho K., Kobuke K., Kishimoto S. (2023). Genetic Risk of Primary Aldosteronism and Its Contribution to Hypertension: A Cross-Ancestry Meta-Analysis of Genome-Wide Association Studies. Circulation.

[103] Goodchild E., Wu X., Senanayake R., MacFarlane J., Argentesi G., Laycock K., Bashari W.A., Cabrera C.P., O'Toole S.M., Salsbury J. (2025). Molecular Imaging Versus Adrenal Vein Sampling for the Detection of Surgically Curable Primary Aldosteronism : A Prospective Within-Patient Trial. Ann. Intern. Med..

[104] Ding J., Li X., Liu S., Gao Y., Zheng G., Hacker M., Zhang Y., Tong A., Huo L. (2024). Clinical Value of (68)Ga-Pentixafor PET/CT in Subtype Diagnosis of Primary Aldosteronism Patients with Adrenal Micronodules. J. Nucl. Med..

[105] Zuo R., Liu S., Ren X., Li W., Xia Z., Xu L., Pang H. (2025). Clinical Utility of Dual-Time (68)Ga-Pentixafor PET/CT in Diagnosing and Subtyping Primary Aldosteronism. Clin. Endocrinol..

[106] Kaneko H., Umakoshi H., Ogata M., Wada N., Ichijo T., Sakamoto S., Watanabe T., Ishihara Y., Tagami T., Iwahashi N. (2022). Machine learning-based models for predicting clinical outcomes after surgery in unilateral primary aldosteronism. Sci. Rep..

[107] Turcu A.F., Freeman M.W., Bancos I., Ben-Shlomo A., Hamidi O., Hamrahian A.H., Huang W., Kirschner L.S., Sam R., Mallappa A. (2025). Phase 2a Study of Baxdrostat in Primary Aldosteronism. N. Engl. J. Med..

[108] Carling T., Scholl U.I. (2025). More about the Phase 2a Study of Baxdrostat in Primary Aldosteronism. N. Engl. J. Med..

[109] Marzano L., Zoccatelli F., Pizzolo F., Friso S. (2025). Adrenalectomy Versus Medical Therapy in Primary Aldosteronism: A Meta-Analysis of Long-Term Cardiac Remodeling and Function: Medical versus Adrenalectomy Treatment Compared in Hyperaldosteronism (MATCH) Study. Hypertension.

[110] Goh J.S., Sohail S., Ayub H., Cheema Z.Z., Paray N.B., Adikari S., Mesmar A., Atout M., Qazi A.R., Aldalqamouni A. (2025). Efficacy and Safety of Aldosterone Synthase Inhibitors in Hypertension: A Systematic Review and Meta-Analysis. Endocrinol. Diabetes Metab..

[111] Braun L.T., Reincke M. (2020). What is the role of medical therapy in adrenal-dependent Cushing's syndrome?. Best Pract. Res. Clin. Endocrinol. Metab..

[112] Carling T. (2025). Adrenalectomy is underused: a call to reduce mortality. Trends Endocrinol. Metab..

[113] Chan C.K., Yang W.S., Lin Y.H., Chang C.C., Wu V.C., Chueh J.S. (2025). KCNJ5 Somatic Mutations Are Associated With Better Long-term Outcomes in Patients With Unilateral Primary Aldosteronism. J. Clin. Endocrinol. Metab..

[114] Grundy M.C., Leung A.A., Pasieka J.L., Harvey A., So C.B., Caughlin C.E., Przybojewski S.J., Orton D.J., Hyrcza M., Kline G.A. (2025). Outcomes After Unilateral Adrenalectomy in Asymmetrical Bilateral Primary Aldosteronism. Hypertension.

